# Supplementary material for: Metatranscriptomic analysis indicates prebiotic effect of isomalto/malto-polysaccharides on human colonic microbiota in-vitro
Source: Sci Rep. 2024 Aug 14;14:18866. doi: 10.1038/s41598-024-69685-w (PMC11324910; doi:10.1038/s41598-024-69685-w)
Supplement: Supplementary file 1 — Supplementary Information. [file 41598_2024_69685_MOESM1_ESM.docx]

**Supplementary material**

**Metatranscriptomic analysis indicates prebiotic effect of Isomalto/malto-polysaccharides on human colonic microbiota *in-vitro***

Klaudyna Borewicz^1, §,*^, Bastian Hornung^1,2, §,#^, Fangjie Gu^3^, Pieter H. van der Zaal^4^, Henk A. Schols^3^, Peter J. Schaap^2^, Hauke Smidt^1^

Supplementary Material

Table S1. Overview over the RNA-seq metrics. The first column indicates the sample name, followed by the total number of sequenced reads, the total number and percentage of identified rRNA reads, and the total amount of non rRNA reads (that is total reads minus rRNA reads). The columns afterwards include the number and percentage of bases trimmed due to adapters, number and percentage of reads passing the quality filtering, their mean length and the percentage of reads which passed all filtering steps. The reads were afterwards mapped to the metatranscriptome assembly, and the percentage of mapping reads is indicated in the last column.

| Condition | total reads | rRNA | % rRNA | non rRNA | trimmed bases due to adapters | % of bases trimmed due to adapters | sequences passing prinseq quality filtering | % passing prinseq quality filtering | mean length | Total % of bases passing ALL filtering steps | Mapping rate in % to assembly |
| --- | --- | --- | --- | --- | --- | --- | --- | --- | --- | --- | --- |
| Blank, repl. 1, t0 | 17182356 | 388147 | 2,26 | 16794209 | 713631983 | 28,14 | 15120013 | 90,03 | 108,34 | 63,56 | 79,06 |
| Blank, repl. 2, t0 | 12843968 | 234407 | 1,83 | 12609561 | 620076640 | 32,57 | 11339128 | 89,92 | 101,95 | 60,00 | 75,99 |
| IMMP-27, repl. 1, t0 | 18661592 | 402676 | 2,16 | 18258916 | 844672334 | 30,64 | 16454635 | 90,12 | 104,71 | 61,55 | 71,29 |
| IMMP-27, repl. 2, t0 | 21152485 | 438493 | 2,07 | 20713992 | 881059209 | 28,17 | 18728000 | 90,41 | 108,06 | 63,78 | 74,42 |
| IMMP-94, repl. 1, t0 | 30405866 | 555904 | 1,83 | 29849962 | 1087733238 | 24,13 | 26908763 | 90,15 | 113,98 | 67,25 | 79,06 |
| IMMP-94, repl. 2, t0 | 19354896 | 777385 | 4,02 | 18577511 | 588584413 | 20,98 | 16575459 | 89,22 | 119,53 | 68,24 | 80,79 |
| Blank, repl. 1, t24 | 27195831 | 243329 | 0,89 | 26952502 | 917731871 | 22,55 | 24835747 | 92,15 | 114,96 | 69,99 | 81,28 |
| Blank, repl. 2, t24 | 26279510 | 263802 | 1,00 | 26015708 | 1199020108 | 30,52 | 23724640 | 91,19 | 103,99 | 62,59 | 81,43 |
| IMMP-27, repl. 1, t24 | 12540536 | 1832716 | 14,61 | 10707820 | 587094636 | 36,31 | 9467522 | 88,42 | 93,59 | 47,10 | 85,32 |
| IMMP-27, repl. 2, t24 | 23402662 | 2094144 | 8,95 | 21308518 | 775851341 | 24,11 | 18915069 | 88,77 | 111,03 | 59,83 | 86,95 |
| IMMP-94, repl. 1, t24 | 14390413 | 901227 | 6,26 | 13489186 | 729841441 | 35,83 | 12186486 | 90,34 | 96,64 | 54,56 | 85,41 |
| IMMP-94, repl. 2, t24 | 27352727 | 1655263 | 6,05 | 25697464 | 1235521747 | 31,84 | 23084321 | 89,83 | 102,74 | 57,80 | 84,52 |
| Blank, repl. 1, t48 | 22532893 | 822320 | 3,65 | 21710573 | 1368385387 | 41,74 | 19533106 | 89,97 | 88,47 | 51,13 | 79,64 |
| Blank, repl. 2, t48 | 23432237 | 902055 | 3,85 | 22530182 | 1200860127 | 35,3 | 20328428 | 90,23 | 97,88 | 56,61 | 80,17 |
| IMMP-27, repl. 1, t48 | 24004294 | 4361225 | 18,17 | 19643069 | 1175519132 | 39,63 | 16574957 | 84,38 | 92,59 | 42,62 | 81,24 |
| IMMP-27, repl. 2, t48 | 21275156 | 8177863 | 38,44 | 13097293 | 691799504 | 34,98 | 11702497 | 89,35 | 98,7 | 36,19 | 80,22 |
| IMMP-96, repl. 1, t48 | 28735621 | 11137710 | 38,76 | 17597911 | 1296700269 | 48,8 | 14765189 | 83,9 | 81,2 | 27,82 | 83,92 |
| IMMP-96, repl. 2, t48 | 35679453 | 11638379 | 32,62 | 24041074 | 1511937150 | 41,65 | 20304145 | 84,46 | 92,59 | 35,13 | 85,36 |
| IMMP-96, repl. 1, t6 | 18066769 | 185180 | 1,03 | 17881589 | 390649819 | 14,47 | 15736624 | 88 | 126,02 | 73,18 | 75,76 |
| IMMP-96, repl. 2, t6 | 20993700 | 55456 | 0,26 | 20938244 | 412525567 | 13,05 | 18327046 | 87,53 | 128,66 | 74,88 | 71,55 |
| IMMP-96, repl. 1, t12 | 35944607 | 70088 | 0,20 | 35874519 | 1270799851 | 23,46 | 31773426 | 88,57 | 113,35 | 66,80 | 76,36 |
| IMMP-96, repl. 2, t12 | 22773537 | 64983 | 0,29 | 22708554 | 964291453 | 28,12 | 20307424 | 89,43 | 106,59 | 63,37 | 76,79 |
| IMMP-96, repl. 1, t24 | 17241186 | 1380292 | 8,01 | 15860894 | 385783659 | 16,11 | 13984773 | 88,17 | 122,24 | 66,10 | 87,1 |
| IMMP-96, repl. 2, t24 | 19319950 | 1502708 | 7,78 | 17817242 | 607265419 | 22,57 | 15673285 | 87,97 | 111,85 | 60,49 | 86,3 |
| IMMP-96, repl. 1, t48 | 34060532 | 145402 | 0,43 | 33915130 | 1420500568 | 27,74 | 30260173 | 89,22 | 107,64 | 63,75 | 75,81 |
| IMMP-96, repl. 2, t48 | 31992519 | 119756 | 0,37 | 31872763 | 1450909006 | 30,15 | 28116504 | 88,21 | 105,1 | 61,58 | 72,91 |
| Blank, repl. 1, t0 | 14780189 | 291372 | 1,97 | 14488817 | 458214833 | 20,94 | 12514201 | 86,37 | 111,32 | 62,84 | 84,1 |
| IMMP-dig27, repl. 1, t6 | 21177851 | 113633 | 0,54 | 21064218 | 1266290906 | 39,81 | 17927736 | 85,11 | 89,44 | 50,48 | 81,34 |
| IMMP-dig27, repl. 2, t6 | 14730499 | 91528 | 0,62 | 14638971 | 464265511 | 21 | 12827318 | 87,62 | 113,79 | 66,06 | 81,04 |
| IMMP-dig27, repl. 1, t12 | 19229982 | 120092 | 0,62 | 19109890 | 353792739 | 12,26 | 16872478 | 88,29 | 128,38 | 75,09 | 81,28 |
| IMMP-dig27, repl. 2, t12 | 19183847 | 184071 | 0,96 | 18999776 | 449569556 | 15,67 | 16837679 | 88,62 | 123,83 | 72,46 | 81,79 |
| IMMP-dig27, repl. 1, t24 | 19000262 | 346531 | 1,82 | 18653731 | 573909821 | 20,38 | 16612325 | 89,06 | 117,09 | 68,25 | 82,71 |
| IMMP-dig27, repl. 2, t24 | 18981915 | 260033 | 1,37 | 18721882 | 214922072 | 7,6 | 16320453 | 87,17 | 135,84 | 77,86 | 83,29 |
| IMMP-dig27, repl. 1, t48 | 19947312 | 1144473 | 5,74 | 18802839 | 508216740 | 17,9 | 16742425 | 89,04 | 120,37 | 67,35 | 88,6 |
| IMMP-dig27, repl. 2, t48 | 11159596 | 475984 | 4,27 | 10683612 | 160119185 | 9,93 | 9455765 | 88,51 | 132,49 | 74,84 | 89,42 |
| Average | 21366411,23 | 1514013,714 | 6,33 | 19852397,51 | 801840435,8 | 25,74 | 17591935,06 | 85,99 | 106,19 | 60,89 | 78,66 |
| Total | 747824393 | 52990480 |  | 694833913 | 28064415252 |  | 615717727 |  |  |  |  |

Table S3. Assignment of genus-level taxa per cluster, showing the amount of assigned genes and differentially expressed genes over all conditions. The clusters were assigned based on the co-expression level of the contigs. Afterwards the number of genes belonging to the relevant organism was counted, and how many were differentially expressed.

| **Organism** | **Genes assigned** | **Genes differentially expressed** |
| --- | --- | --- |
| **Cluster 1** |  |  |
| *Ruminococcus* | 9904 | 6220 |
| *Lactococcus* | 8211 | 5263 |
| unclassified*_*Gammaproteobacteria | 373 | 285 |
| Bos | 329 | 263 |
| N/A | 208 | 85 |
| unclassified_Mammalia | 198 | 67 |
| unclassified_Bovidae | 107 | 41 |
| Clostridiales | 4 | 0 |
| Bacteria | 3 | 1 |
| Eukaryota | 2 | 1 |
| Gammaproteobacteria | 1 | 1 |
| Viruses | 1 | 0 |
| **Cluster 2** |  |  |
| *Lactobacillus* | 8655 | 7279 |
| *Bifidobacterium* | 7817 | 5849 |
| unclassified_Actinobacteria | 265 | 195 |
| unclassified_Bifidobacteriaceae | 116 | 98 |
| *Fusobacterium* | 70 | 47 |
| unclassified_Lactobacillaceae | 52 | 38 |
| Myoviridae | 36 | 33 |
| **Cluster 3** |  |  |
| *Enterococcus* | 2580 | 1927 |
| unclassified_Lactobacillales | 1154 | 1012 |
| unclassified_Bacilli | 537 | 473 |
| unclassified_Enterococcaceae | 139 | 136 |
| **Cluster 4** |  |  |
| unclassified_Enterobacteriaceae | 11350 | 9255 |
| unclassified_Bacteria | 6997 | 4494 |
| *Eubacterium* | 4884 | 3849 |
| *Escherichia* | 2972 | 2386 |
| unclassified*_*Proteobacteria | 683 | 530 |
| *Salmonella* | 59 | 47 |
| *Shigella* | 51 | 9 |
| *Enterobacter* | 36 | 29 |
| *Citrobacter* | 31 | 14 |
| *Vibrio* | 26 | 9 |
| **Cluster 5** |  |  |
| *Bacteroides* | 53749 | 49101 |
| unclassified*_*Bacteroidales | 10243 | 9067 |
| *Parabacteroides* | 1749 | 919 |
| *Prevotella* | 302 | 233 |
| *Bacteria* | 193 | 133 |
| *Desulfosporosinus* | 32 | 11 |
| *Flavobacterium* | 30 | 27 |
| **Cluster 6** |  |  |
| *Clostridium* | 4244 | 3228 |
| unclassified*_*Clostridia | 114 | 69 |
| **Cluster 7** |  |  |
| unclassified_Clostridiales | 10819 | 3356 |
| unclassified_Lachnospiraceae | 1030 | 219 |
| *Anaerostipes* | 127 | 6 |
| Clostridiales | 39 | 5 |
| **Cluster 8** |  |  |
| N/A | 8225 | 4083 |
| *Sutterella* | 3598 | 2645 |
| unclassified_Bacteroidetes | 770 | 681 |
| unclassified_Betaproteobacteria | 135 | 77 |
| *Odoribacter* | 94 | 76 |
| *Ethanoligenens* | 28 | 21 |
| *Corynebacterium* | 20 | 8 |
| **Cluster 9** |  |  |
| *Bilophila* | 3853 | 2614 |
| unclassified*_*Firmicutes | 3152 | 1898 |
| *Phascolarctobacterium* | 1328 | 8 |
| unclassified*_*Selenomonadales | 244 | 2 |
| *Acidaminococcus* | 174 | 4 |
| unclassified*_*Acidaminococcaceae | 117 | 0 |
| *Selenomonas* | 109 | 19 |
| *Veillonella* | 88 | 8 |
| *Megamonas* | 64 | 12 |
| unclassified*_*Veillonellaceae | 56 | 3 |
| *Pelosinus* | 53 | 4 |
| *Megasphaera* | 53 | 9 |
| *Desulfitobacterium* | 51 | 15 |
| *Anaeromusa* | 33 | 0 |
| *Acetonema* | 32 | 3 |
| *Mitsuokella* | 20 | 5 |

**Supplementary Methods**

Text mining

Further EC numbers were derived by text mining and matching all InterProScan derived domain names against the BRENDA database (download 13.06.13) [23]. The text mining algorithm included lower casing all characters, removal of non-alphanumerical characters (colons, commas, brackets, apostrophes, dashes, terminal points), removal of partial and generic terms (type, terminal, subunit, domain, enzyme, like, hypothetical, conserved, operon, active site, enzyme, probably, central, 51 kd, respiratory chain, c terminal, n terminal), rejection of overly generic final result terms (kinase, cytochrome, protein, methyltransferase) and reduction of certain terms (deletion of PEP/pyruvate binding; removal of “prokaryotic” in “prokaryotic cytidylate kinase”; “family” in “cytidilate kinase family”; “phosphorylating” in “glyceraldehyde phosphate dehydrogenase phosphorylating”; “iron containing” in “iron containing alcohol dehydrogenase”; “zinc containing” in “zinc containing alcohol dehydrogenase”; “manganese containing” in “manganese containing catalase”; “20 kd” in “nadh ubiquinone oxidoreductase 20 kd”; replacement of “carboxyltransferase” with “carboxylase” in “pyruvate carboxyltransferase”). Furthermore, all terms, which were only of length one, were also removed, in case the remaining name contained more than two words. On some domain names a manual curation was performed, and overly generic identifications (e.g. matching PF12847 “Methyltransferase domain” with e.g. EC 2.1.1.124 with alternative name “Protein Methyltransferase I”) were rejected.

**Supplementary Figures**

Figure S1: Experimental design. Two different batches of fermentation were performed. The first batch contained two replicates without any added carbohydrate, two replicates with IMMP27 and two replicates with IMMP97, which were sequenced at 0h of fermentation, 24h and 48h. The second batch contained two replicates with pretreated IMMP27 and IMMP95, which were sequenced at 6h, 12h, 24h and 48h. Ribosomal RNA was removed with the RiboZero kit, and the RNA was afterwards sequenced on an Illumina HiSeq machine. The remaining RNA was filtered computationally, adapters were removed, and quality trimming was performed. Afterwards all reads were pooled and one assembly was produced. All reads were mapped to this assembly to determine expression. Taxonomy was assigned to the contigs, and functions to genes. This information was used for further metabolic mapping. A symbolic pathway with different types of genes of different lengths are depicted, producing a compound H from a starting compound A.

Figure S2. Relative abundance (percentage) of starch and sucrose metabolism enzyme encoding genes detected in the metatranscriptome data. These numbers represent the combined expression of enzymes depicted in figure 4.

Figure S3. Heatmap of log10 transformed relative abundances of expressed genes detected in our data coding for starch and sucrose metabolism enzymes. Samples clustered based on the similarities between the up and down regulated genes, as depicted in the hierarchical clustering on top of the figure. The red arrows indicate selected genes that code for enzymes described in our IMMP degradation model. Green boxes highlight the gene upregulation patterns for different IMMPs at various incubation times. The x-axis shows the sample names. The y-axis shows the enzyme, based on EC classification.

Figure S4. IMMP degradation model. A. main enzymes involved in the pathway, B. relative abundance of transcripts of genes coding for enzymes needed for IMMP degradation.

Figure S5. Relative contribution of different bacterial groups to expression of genes coding for the enzymes in the IMMP degradation pathway. Four identical schemes are presented for the four different substrates, with the pie charts representing the contribution of varying bacterial groups to each step. The numbers on the arrows represent enzyme commission numbers for the respective steps.

Figure S6. Volcano plot of all comparisons which are considered in this article. Genes with a statistically significant difference in expression with a multi-test corrected p-value (q-value) <0.01 are depicted in orange, non-significant genes in grey. The number of significant and non-significant genes is mentioned below each plots. Total numbers differ, as not all genes are expressed in each comparison. The x-axis of each plot shows the log10 difference in abundance between the two tested conditions. The y-axis shows the logarithm of the q-value, multiplied by -1.

Figure S7: Overview over the clustering procedure. First, expression was lumped at the genus level. On the accumulated expression data k-means clustering was performed, until a stable clustering was achieved. The genes of the grouped genera were afterwards subjected to DBSCAN clustering. The stability of the clustering was evaluated with the Tau-parameter. Only genes, which were at least once differentially expressed, were used in the clustering process to reduce the noise.
