# Supplementary figures and images for: Metatranscriptomic analysis indicates prebiotic effect of isomalto/malto-polysaccharides on human colonic microbiota in-vitro
Source: Sci Rep. 2024 Aug 14;14:18866. doi: 10.1038/s41598-024-69685-w (PMC11324910; doi:10.1038/s41598-024-69685-w)

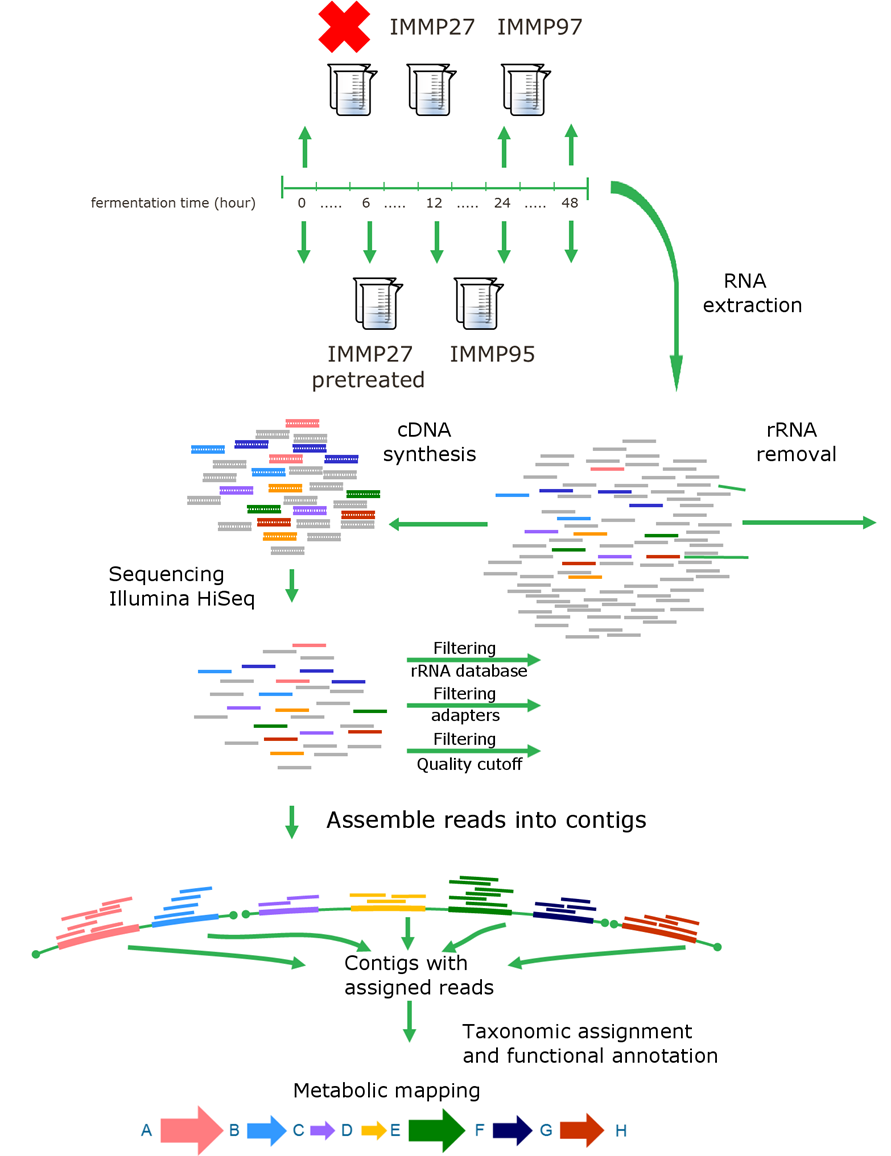

Supplement: Supplementary file 2 — Supplementary Figure S1. [file 41598_2024_69685_MOESM2_ESM.tif]

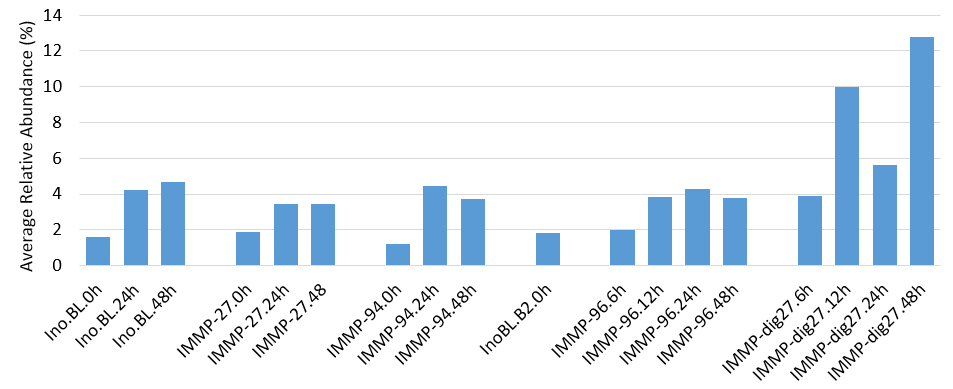

Supplement: Supplementary file 3 — Supplementary Figure S2. [file 41598_2024_69685_MOESM3_ESM.png]

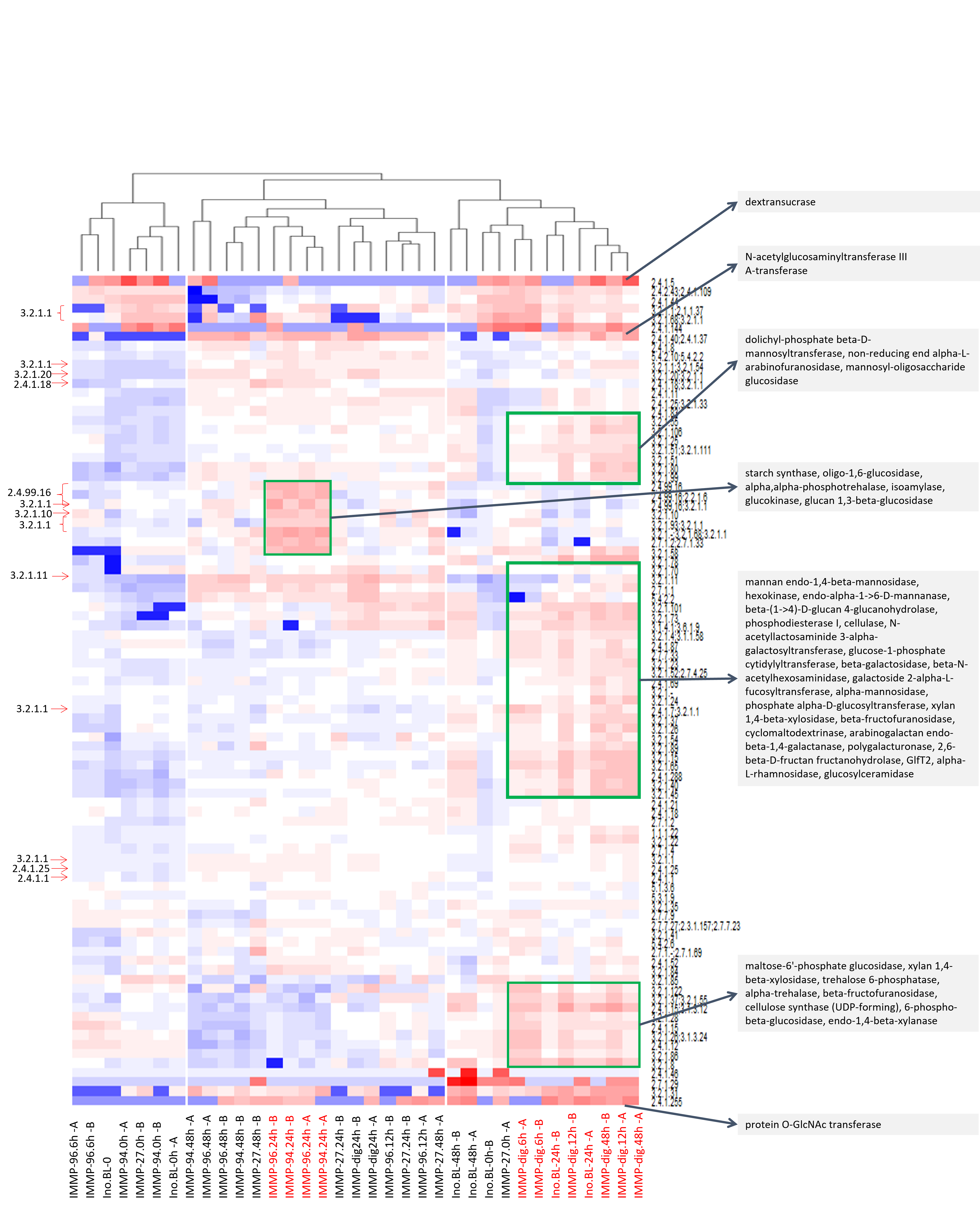

Supplement: Supplementary file 4 — Supplementary Figure S3. [file 41598_2024_69685_MOESM4_ESM.png]

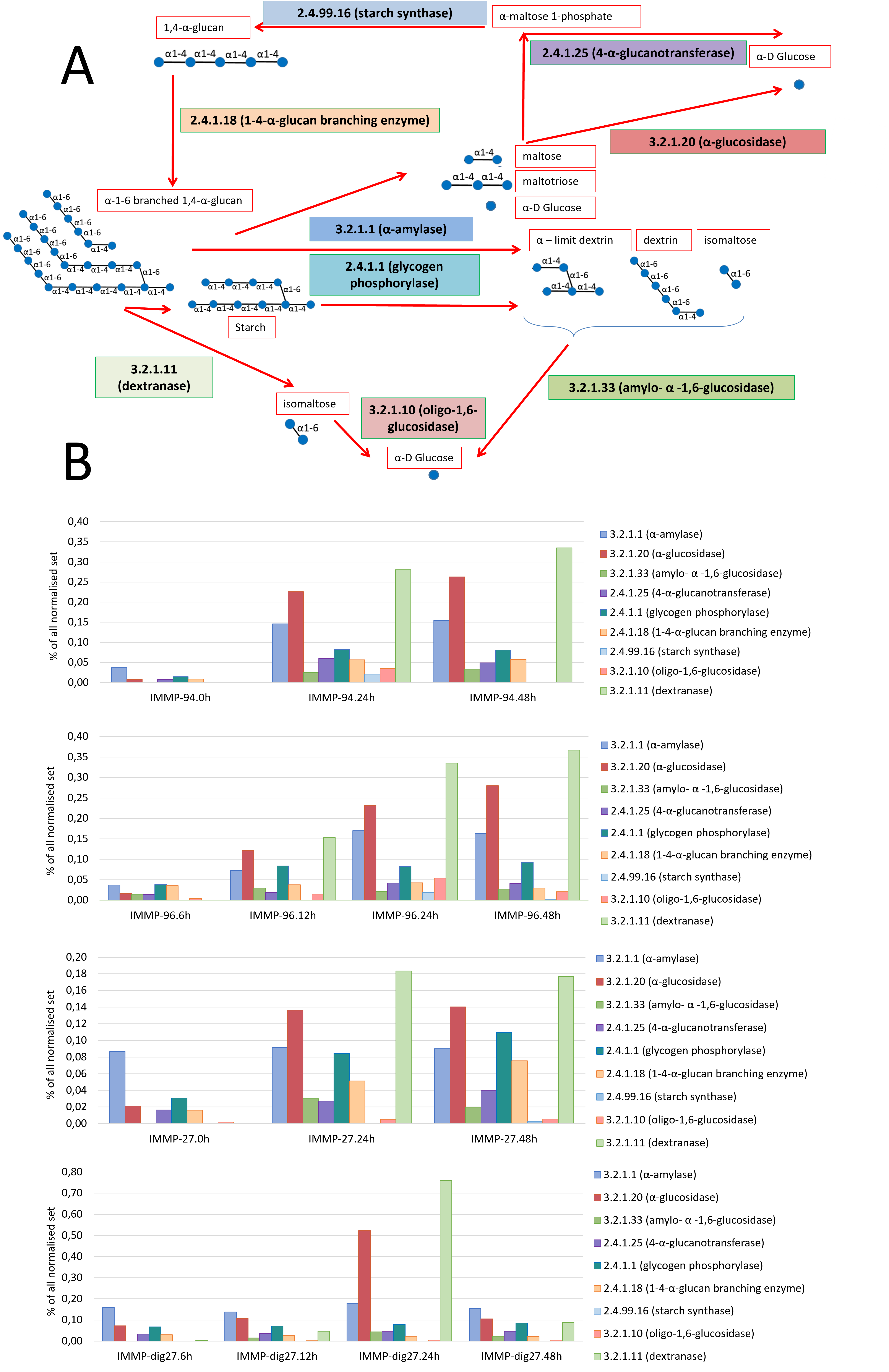

Supplement: Supplementary file 5 — Supplementary Figure S4. [file 41598_2024_69685_MOESM5_ESM.png]

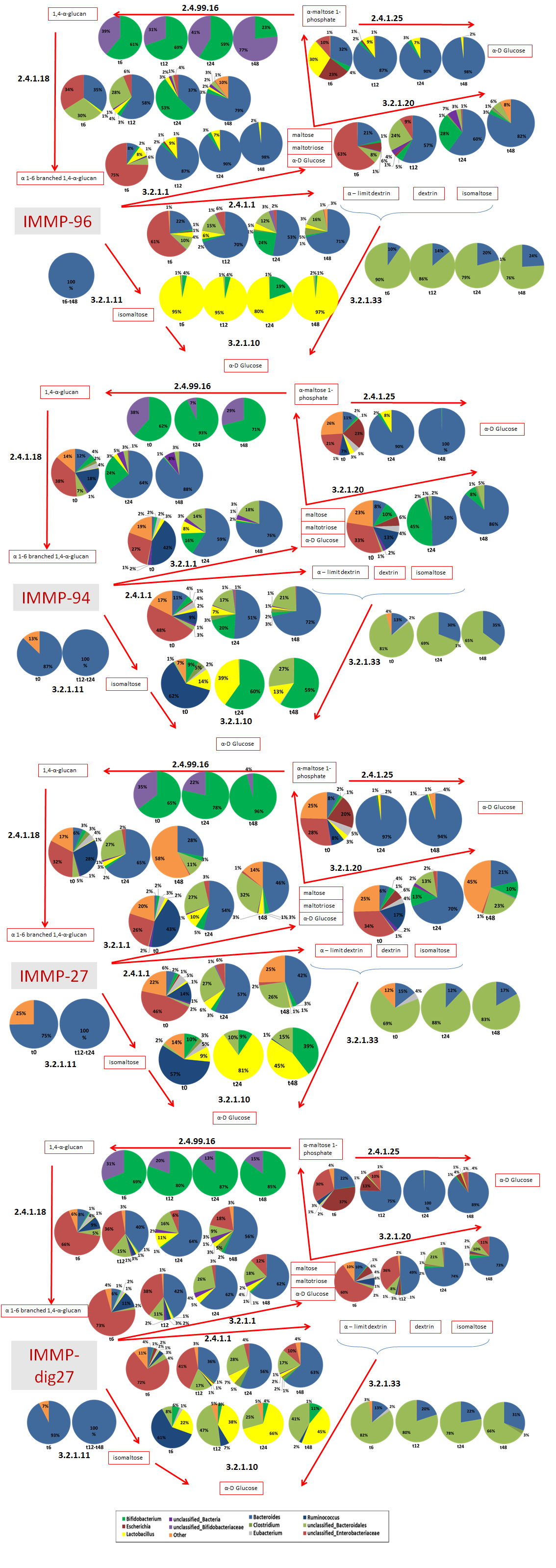

Supplement: Supplementary file 6 — Supplementary Figure S5. [file 41598_2024_69685_MOESM6_ESM.tif]

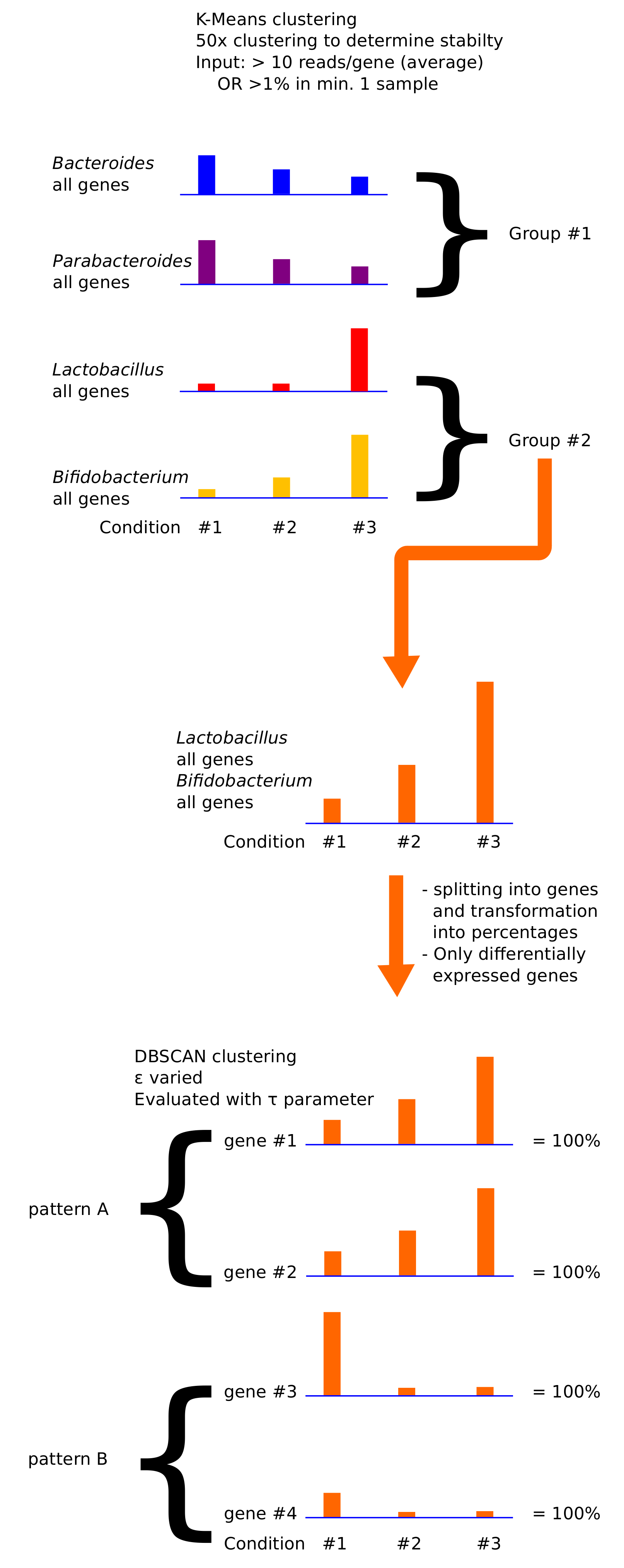

Supplement: Supplementary file 8 — Supplementary Figure S7. [file 41598_2024_69685_MOESM8_ESM.tif]
